# High-Density Lipoprotein Correlates with Cognitive Functioning in Schizophrenic Women

**DOI:** 10.3390/brainsci14070699

**Published:** 2024-07-12

**Authors:** Maria Staniek, Pawel Kapelski, Przemyslaw Zakowicz, Aleksandra Rajewska-Rager, Karolina Wasicka-Przewozna, Maria Skibinska

**Affiliations:** 1 Independent Researcher, 60-806 Poznan, Poland; 2 Department of Psychiatric Genetics, Poznan University of Medical Sciences, 60-806 Poznan, Poland; 3 Collegium Medicum, University of Zielona Góra, 65-417 Zielona Gora, Poland

**Keywords:** schizophrenia, cognitive functions, Wisconsin card sorting test (WCST), positive and negative syndrome scale (PANSS), lipid profile, low-density lipoprotein (LDL), high-density lipoprotein (HDL), triglycerides (TGs)

## Abstract

(1) Background: Schizophrenia is a chronic and progressive neuropsychiatric illness. Apart from positive and negative symptoms, 98% of the population diagnosed with schizophrenia have impaired cognitive functioning, which significantly influences the quality of life. The correlation between lipids and cognitive functioning has been well established. Our study aimed to investigate correlations between cognitive functions, the severity of schizophrenia symptoms, and lipid profiles. (2) Methods: Fifty-two women diagnosed with schizophrenia participated in this study. Cognitive functioning was measured using the Wisconsin Card Sorting Test (WCST). The Positive and Negative Symptom Scale (PANSS) was used. The serum lipid profile, including low-density lipoproteins (LDLs), high-density lipoproteins (HDLs), and triglycerides was measured. (3) Results: Better cognitive functions were associated with normal HDL levels, while low HDL levels correlated with worse WSCT scores. Only the PANSS negative subscale showed a correlation with HDL levels. Correlations with chronicity of schizophrenia and the patient’s age with poorer cognitive functions, but not with symptom severity, were detected. Early/late age at onset did not influence WSCT scores. (4) Conclusions: Our results suggest high HDL levels might be a protective factor against cognitive impairment. The influences of age and illness duration also play a vital role in cognitive performance.

## 1. Introduction

Approximately 1% of the population worldwide is afflicted with schizophrenia [1]. In accordance with data from the Global Burden of Disease, the prevalence of schizophrenia has been increasing since 1990 [2]. Schizophrenia is a chronic and progressive neuropsychiatric illness, which constitutes symptoms conventionally divided into two groups: positive and negative; in recent years, the role of a third group of symptoms, cognitive deficits, has increased significantly due to its influence on daily functioning and the quality of life of patients [3,4,5]. A total of 98% of the population diagnosed with schizophrenia have impaired cognitive functioning [6]. Treatment of cognitive dysfunctions may prove to be essential in hindering the progression of the disease leading to disability, of which schizophrenia is a sizable contributor worldwide [5,7,8].

Commonly found neurocognitive deficits in schizophrenia are processing speed, attention, working memory, problem-solving, verbal memory, and learning, as well as visual learning [5,9,10]. Studies show that cognitive dysfunctions are present during the first episode of the illness [11,12,13], as well as during its course [14], some of them suggest that even before the onset of the disease, cognitive symptoms are already in existence [15,16].

The correlation between lipids and cognitive functioning has been well established. Although the blood–brain barrier obstructs the free flow of lipids [17], the changes in serum cholesterol levels still influence brain cholesterol content [18]. Studies conducted on animals showed that learning and memory impairment reversed when adding cholesterol to the diet of animals with cholesterol deficiency and animals with a cholesterol synthesis block [19,20]. Furthermore, they proved a connection between a cholesterol-enriched diet and improved results in memory acquisition and retention, as well as performance [21,22]. The results in the human population are inconsistent. On the one hand, Elias et al. found a strong correlation between low total serum cholesterol and worse scores for cognitive functions such as abstract reasoning, attention, word fluency, and cognitive control [23]. Moreover, introducing treatments aiming at lowering cholesterol levels, both pharmacological [24] and dietary ones [25], worsened some of the cognitive measures. However, other publications suggest a negative correlation between cognitive functions and total serum cholesterol levels [26]. Meanwhile, Okusaga et al. reported no significant links between cholesterol levels and cognition [27].

Cognitive impairment is a primary determinant of functional disability and the indirect costs of schizophrenia. Pharmacological treatment options for cognitive deficits are unsatisfactory due to limited efficacy or tolerability. In the past, the main focus of treatment response was positive symptoms. At the same time, less attention was paid to negative symptoms and cognitive impairment, considering it as the “residual phase” of illness. In recent years, there has been increasing interest in assessing functional improvement in the remission of positive symptoms. Non-pharmacological interventions, such as cognitive remediation, suggest potential benefits. The augmentation of antipsychotic treatment with pharmacological cognitive enhancers has been studied, with a lack of evidence in the long term. Combined treatment approaches appear particularly promising, potentially significantly increasing the percentage of treatment responders [28].

### The Aim of the Study

In this paper, we plan to underline the links between cholesterol levels and neurocognitive deficits in schizophrenic patients, using the WCTS as a measurement method. Additionally, we will show associations between negative and positive symptoms during an acute episode and in remission, using PANSS results and cognitive functioning. Furthermore, we will investigate the correlations between cognitive impairment and the severity of positive and negative symptoms of schizophrenia in relation to biomedical variables such as age, age of onset, and illness duration.

## 2. Materials and Methods

### 2.1. Participants

Fifty-two women (mean age 32.96, SD 10.9) diagnosed with schizophrenia were recruited from 2008–2015 at the Department of Psychiatry, Poznan University of Medical Sciences, Poland. All subjects were of Caucasian origin, and they were the native Polish population from the Great Poland region. This study was performed in accordance with the ethical standards established in the Declaration of Helsinki and was approved by the medical ethics committee of the Poznan University of Medical Sciences (approval no 266/08). All participants gave written informed consent before being included in this study, and their anonymity was preserved. During recruitment, male patients often refused to participate in the study, resulting in a significant disproportion between females and males. Therefore, in the present study, we decided to include only women to improve the study group homogeneity.

### 2.2. Clinical Assessment

Participants were recruited during the exacerbation of psychotic symptoms, within a week from admission to the hospital ward. The inclusion criteria included the exacerbation of psychotic symptoms at hospital admission (T0), a diagnosis of schizophrenia according to DSM-IV criteria, and being aged between 18–65 years. The exclusion criteria included chronic or acute somatic or neurological diseases, pregnancy or breastfeeding, and increased CRP (C-reactive protein). A consensus diagnosis of schizophrenia was made by two experienced psychiatrists for each patient using the Structured Clinical Interview for DSM-IV Axis I Disorders (SCID) [29]. Patients were evaluated also for lifetime psychiatric symptomatology with the Operational Criteria for Psychotic Illness (OPCRIT) [30]. The Positive and Negative Syndrome Scale (PANSS) [31] was used to assess the severity of schizophrenia symptoms at admission (T0) and after eight weeks of treatment (T8). The PANSS is a 30-item semi-structured scale used to determine the severity of schizophrenia symptoms. Each item is rated on a Likert scale from 1 (absent) to 7 (extreme). It consists of three subscales: positive (PANSS P) and negative (PANSS N), each with seven items, and general psychopathology (PANSS G), with sixteen items. The total score on the scale is also evaluated (PANSS total).

A computer version 4 of the Wisconsin Card Sorting Test (WCST) was given to the patients in the remission of psychotic symptoms, after 8 weeks of hospitalization. We analyzed all the domains of the WCST, but only a percentage (%) of errors, perseverative responses, perseverative errors, nonperseverative errors, and conceptual level responses are presented in the article [32].

### 2.3. Lipid Profile

The fasting blood lipid profile, including low-density lipoproteins (LDLs), high-density lipoproteins (HDLs), and triglycerides (TGs), were measured at admission to the psychiatric ward (T0), and after 8 weeks of treatment (T8) using standard laboratory procedures, on a Beckman Coulter AU 680 biochemistry analyzer (Beckman Coulter, Inc., Brea, CA, USA). The Polish lipid norm cut-offs are LDL < 115 mg/dL, HDL > 50 mg/dL, and TG < 150 mg/dL.

### 2.4. Statistical Analyses

The Kolmogorov–Smirnov and Shapiro–Wilk tests were used to test the normality of the data. Most of the data showed non-normal distribution, thus non-parametric statistical tests were applied in the analyses, including the Mann–Whitney U-test, Wilcoxon test, and Spearman’s correlation. The statistical significance level was set at *p* < 0.05. Statistical analyses were conducted in Statistica v13 software.

## 3. Results

### 3.1. Demographic Characteristic

We enrolled 52 women (mean age, 32.96 (±10.90) years) diagnosed with schizophrenia in this study, with a mean age at onset of 24.85 (±6.50) years and a mean illness duration of 8.12 (±8.69) years. In our group, patients with recent onset (≤5 years) schizophrenia constituted 58% (*n* = 30), while the chronic (>5 years) schizophrenia group was represented by 42% (*n* = 22) patients. Pharmacological treatment was as follows: three patients (*n* = 3) were medicated exclusively with a typical antipsychotic (haloperidol), and two of them with adjuvant benzodiazepines. Fourteen participants (*n* = 14) were treated with a typical antipsychotic (haloperidol) with adjuvant atypical neuroleptics: olanzapine (*n* = 7), clozapine (*n* = 4), ziprasidone (*n* = 2), risperidone (*n* = 2), and aripiprazole (*n* = 1). Other patients were treated with atypical neuroleptics: olanzapine (*n* = 15), clozapine (*n* = 9), aripiprazole (*n* = 6), amisulpride (*n* = 4), ziprasidone (*n* = 3), risperidone (*n* = 3), and quetiapine (*n* = 1). Additionally, benzodiazepines (*n* = 16) and antidepressants (*n* = 12) were administered to the patients. Demographic and clinical characteristics of the participants are presented in Table 1.

### 3.2. Changes in Severity of Schizophrenia Symptoms, Lipid Profile, and Weight during Eight Weeks of Treatment

We observed a significant decrease in all subscales of the PANSS after eight weeks of treatment (PANSS P *p* < 0.001, PANSS N *p* < 0.001, PANSS G *p* < 0.001, and PANSS total *p* < 0.001). The serum levels of LDL (*p* = 0.02) and TG (*p* = 0.002) increased during eight weeks of treatment. We did not detect differences in serum HDL (*p* = 0.074) concentrations during eight weeks of hospitalization. We detected a significant increase in weight (*p* < 0.001) during eight weeks of treatment; however, weight gain during treatment did not exceed the BMI norms in patients with BMI ≥ 25.

### 3.3. Comparisons of Wisconsin Card Sorting Test (WCST) Results, Positive and Negative Syndrome Scale (PANSS), and Lipid Profile with Regard to Disease Onset (Recent (≤5 years) vs. Chronic (>5 Years)

During our research, we divided our patients into two groups: recent onset with the duration of the disease ≤5 years, and chronic, who suffered from schizophrenia for longer than five years. We have compared the result of the WCTS of those two fractions and found significant differences in the following psychometric scores: total correct (*p* = 0.003), % perseverative errors (*p* = 0.039), and failure to maintain set (*p* = 0.044). We did not find significant differences in the PANSS at T0 and T8, as well as lipids LDL, HDL, and TG at T0 and T8 when comparing recent onset vs. chronic patients diagnosed with schizophrenia. The results are presented in Table 2.

### 3.4. Comparisons of Wisconsin Card Sorting Test (WCST) Results and Positive and Negative Syndrome Scale (PANSS) with Regard to LDL, HDL, and TG Concentrations (Normal vs. beyond the Norm)

We detected significant differences in WCST results concerning HDL levels: normal levels of HDL corresponded to lower scores of trials administered (*p* = 0.012), % errors (*p* = 0.007), % perseverative errors (*p* = 0.007), and % nonperseverative errors (*p* = 0.004). Higher scores in % conceptual responses (*p* = 0.008) and categories completed (*p* = 0.007) were also noticed in patients with normal HDL levels, while a lower-than-average HDL level was associated with lower scores in these two domains of the WSCT. In our study, patients with higher PANSS negative scores at T8 had low HDL levels (*p* = 0.035). We found no differences in WCST and PANSS scores concerning LDL and TG concentrations. Results are presented in Table 3.

### 3.5. Comparisons of Wisconsin Card Sorting Test (WCST) Results with Regard to Pharmacological Treatment and Smoking Status

Comparing groups of patients treated with clozapine vs. olanzapine we did not reveal any significant differences in WCST performance: trials administered (Z = 1.19, *p* = 0.23), total correct (Z = 0.7, *p* = 48), % errors (Z = 0.81, *p* = 0.42), % perseverative responses (Z = 0.97, *p* = 0.33), % perseverative errors (Z = 1.06, *p* = 0.28), % nonperseverative errors (Z = 0.56, *p* = 0.58), % conceptual level responses (Z = −0.92, *p* = 0.36), categories completed (Z = −49, *p* = 0.62), trials to complete 1st category (Z = 0.32, *p* = 0.75), failure to maintain set (Z = 0.07, *p* = 0.95), and learning to learn (Z = −0.32, *p* = 0.75).

We also did not detect differences in WCST performance with regard to haloperidol, benzodiazepines, nor antidepressants.

Non-smoking patients had a significantly lower % of nonperseverative errors (*p* = 0.022) as well as a higher % of conceptual level responses (*p* = 0.049) compared to smoking patients.

The results are presented in Supplementary Table S1.

### 3.6. Spearman’s Correlation of Wisconsin Card Sorting Test (WCST) Results with Clinical Variables and Lipid Profile

#### 3.6.1. Correlations between Scores on Wisconsin Card Sorting Test (WCST) and Lipid Serum Levels

**Figure 1:**
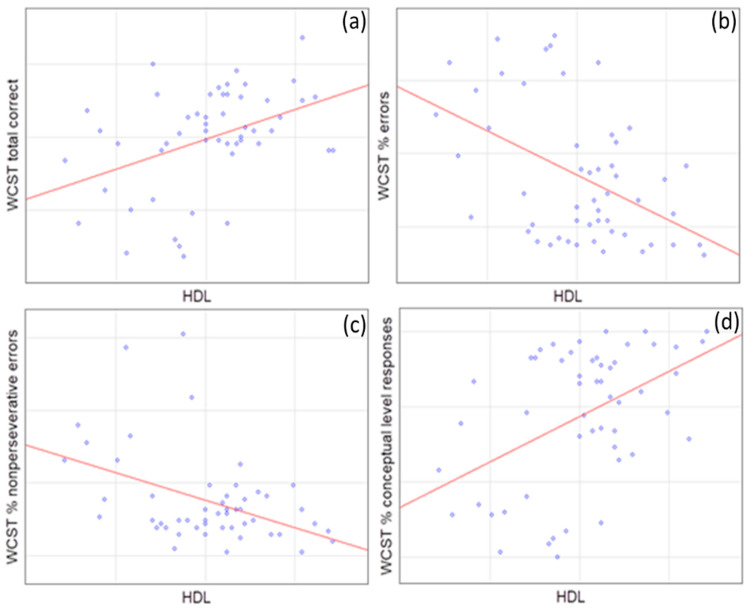
Spearman’s correlations of WCST domains with high-density lipoprotein (HDL) levels. (**a**) WCST total correct (R = 0.38, *p* = 0.005); (**b**) WCST % errors (R = −0.40, *p* = 0.002); (**c**) WCST % nonpersererative errors (R = −0.33, *p* = 0.017); and (**d**) WCST % conceptual level responses (R = 0.41, *p* = 0.003).

In accordance with our findings, HDL levels corresponded positively to the WCST total correct score (*p* = 0.005), as well as the % of conceptual level responses (*p* = 0.003) and WCST categories completed (*p* = 0.015), contrary to the % of errors (*p* = 0.002), the % of nonperseverative errors (*p* = 0.017), and WCST trials to complete the 1st category (*p* = 0.016) to which it corresponds negatively (Figure 1).

LDL levels and WCST learning-to-learn score (*p* = 0.038) were significantly positively correlated. There was no other association between LDL levels and the WCTS, which was statistically significant. Triglycerides (TGs) showed a weak positive correlation with the % of nonperseverative errors (*p* = 0.047). Our research shows no other influence of TG serum levels on cognitive scoring.

#### 3.6.2. Correlations between Scores of Positive and Negative Syndrome Scale (PANSS) and Lipid Serum Levels

The only notable correspondence detected was between negative symptoms PANSS N at T8 and HDL levels (*p* = 0.03). We did not identify any significant links between the severity of positive symptoms, the score of the general psychopathology subscale, or the total score on the PANSS test with lipid serum levels.

#### 3.6.3. Correlations between Scores on Wisconsin Card Sorting Test (WCST) and Positive and Negative Syndrome Scale (PANSS)

Our results show no significant correlation between the severity of schizophrenia symptoms at the beginning of the study (T0) measured with the PANSS scale, PANSS total, PANSS P, PANSS N, and PANSS G, and cognitive functioning after eight weeks of treatment. The apparent associations were detected while comparing PANSS scores obtained at the eight weeks of treatment. We found correlations of WCST results with the severity of negative symptoms (PANSS N) at T8. The higher the score on PANSS N (T8), the more errors the patients made. A significant positive correlation was found with WCST trials administered (*p* = 0.006), the % of total errors (*p* = 0.004), the % of perseverative errors (*p* = 0.043), and the % of nonperseverative errors (*p* = 0.014). A PANSS N score (T8) was negatively associated with the WCST % of conceptual level responses (*p* = 0.005) and categories completed (*p* = 0.024).

We found the general psychopathology subscale of PANSS G at T8 to be positively correlated with the total % of errors made on the WCST (*p* = 0.027), in contrast to the WCST % of conceptual level responses, which was negatively correlated with a PANSS G score (T8) (*p* = 0.384). Positive symptoms (PANSS P at T8) significantly influence WCST failure to maintain set (*p* = 0.013) and the % of nonperseverative errors (*p* = 0.039). A high total score on the PANSS correlated to the severity of cognitive impairment, which was apparent in its positive association between the WCST % of nonperseverative errors (*p* = 0.047).

#### 3.6.4. Correlations between Scores on Wisconsin Card Sorting Test (WCST), PANSS (T8), and Lipid Profile with Age, Age of Onset, and Illness Duration

We found the patient’s age to be a significant variable in scores of cognitive functioning tests. Our research exhibited a positive correlation between age and the % of errors (*p* = 0.036). This is in contrast to the negative connotation between age and a WCTS total correct score (*p* = 0.0004) and conceptual level response (*p* = 0.0009), in addition to categories completed (*p* = 0.037).

The most significant results were noted while comparing the impact of illness duration on cognitive functioning. The scores on the WCST most impaired by the continuing span of the disease were trials administered (*p* = 0.033), the % of error (*p* = 0.002), the % of perseverative responses (*p* = 0.014), the % of perseverative errors (*p* = 0.008), as well as the % of nonperseverative errors (*p* = 0.042). Moreover, negatively correlated with illness duration, WCST psychometric scores were the total correct (*p* = 0.007), the % of conceptual level responses (*p* = 0.003), in addition to categories completed (*p* = 0.003).

We did not detect any notable correlations between the age of onset of schizophrenia and cognitive impairment in the tested sample.

We did not detect any correlations between PANSS scores, low-density lipoprotein (LDL), high-density lipoprotein (HDL), and triglyceride (TG) levels either at T0 or at T8, with age, age at onset, or illness duration.

## 4. Discussion

Our study’s main focus was to determine the correlation between lipid serum levels and cognitive functioning in schizophrenic patients. The available literature presents inconsistent findings in this area of research.

HDL serum levels have been researched in association with cognitive functioning for many years. For example, experiments on animal models of Alzheimer’s disease have proven HDL to be a protective factor of cognition [33,34]. Exploration was also conducted on a healthy population of subjects when it comes to the impact of HDL on cognitive functioning. According to many published researchers, high-density cholesterol serum levels may be treated as a protective factor against cognitive decline [35,36,37,38]. In Azheimer’s disease, HDL levels are lowered, which corresponds to a decrease in MMSE results [39,40]. Whereas, according to the findings of Mehdi et al. (2024), depressive symptoms influenced the protective character of HDL on memory function, thus leading to a worsening of cognitive functions in patients with diagnosed depression [41]. In their paper, Jia et al. (2020) present a positive correlation between HDL levels and cognitive functioning [42]. Furthermore, low serum levels of HDL in bipolar patients corresponded to cognitive deficits [43].

According to our results, higher HDL levels correspond to better cognitive functioning in patients with schizophrenia. Similar findings were reported by Lindenmayer et al. (2012) [44], in contrast to the paper by Liu et al. (2022) in which for the group of patients under 45 years of age, high levels of HDL corresponded to poorer cognition [45].

Other papers produce results of a strong positive correlation between total cholesterol serum levels and cognitive functioning [45,46,47]. The outcome of our research does not support that premise, similar to our conclusions published before [48,49].

Triglyceride serum levels significantly contributed to cognitive functioning in previously published papers [47,48] as a protective factor. Our results cannot support that hypothesis because a significant positive correlation between TG levels was only detected in relation to WCST nonperseverative errors, which did not remain significant after considering laboratory norms of TG serum levels.

The important role of lipids in the pathology underlying schizophrenia and the cognitive impairment that goes with it may be explained by more than one hypothesis. Cholesterol levels can influence the activity of neurotransmitters in the brain. According to Dufour et al. (2006), low cholesterol levels impair glutaminergic synaptic functioning in the hippocampus [21], which is a part of the brain of great importance in short-term memory processing [50]. Decreased cholesterol may also reduce serotonin activity in the brain, influencing the number of serotonin transporters and receptors [51]. Another notifiable theory is the correlation between increased total cholesterol serum levels and increased brain-derived neurotrophic factor (BDNF) in the population of schizophrenic patients [52], which was found to play a role in cognitive impairment [53,54]. Some researchers propose serum cholesterol levels as a representative marker for polyunsaturated fatty acids (PUFAs), which take part in synthesizing neurotransmitters and are involved in neuron membrane formation, thus explaining the correlation between cognitive functioning and cholesterol [55].

The progressive character of cognitive decline of schizophrenia patients has been widely reported throughout time [48,56,57,58]. Our study supports this premise. Illness duration was significantly positively correlated with the number of WCST trials administered, including total amount of error, percentage of error, number and percentage of perseverative response, number and percentage of perseverative errors, and percentage of nonperseverative errors. At the same time, it negatively correlated with the WCST total correct score, and the number and percentage of conceptual level responses, in addition to categories completed. Banno et al. (2012) found a similar association using WCST as a measuring tool for cognitive functioning [58]. We divided our group into two sub-groups depending on the duration of illness: recent onset, with the duration of the disease less than five years, and chronic, who suffered from schizophrenia for longer than five years. We compared the result of the WCTS of those two fractions and found significant associations in the following psychometric scores: total correct, number and percentage of errors, number and percentage of perseverative responses, number and percentage of perseverative errors, and conceptional level responses. However, not all the studies are in agreement with the progressive character of cognitive dysfunction in schizophrenia. Stratta et al. (2004) divided the group of schizophrenic patients into “good” and “poor” performers on the WCST and observed them throughout the years. The results of the sub-group of “poor performers” did not indicate the deteriorating character of schizophrenia. Meanwhile, “good performers” improved their WCST scores in the intermediate length-of-illness group (6–10 years of illness) and then declined in the third one (>10 years) [59].

The age factor on the WCTS result was significant—it was evident that the older patients are, the poorer their cognitive skills. Similar reports have been published before [48,53].

However, the age of onset of schizophrenia was not shown as a risk factor for cognitive deterioration in our study, supporting results presented by Banno et al. (2012) [58]. Our findings differ from those of Bellino et al. (2004) [60].

Our sample consisted only of women. However, the role of gender in the cognitive functioning of patients with schizophrenia remains unclear. Female patients with schizophrenia were found to score worse than males in reasoning, working memory, and problem-solving, according to Pérez-Garza et al. (2016) [61] and Peng et al. (2021) [62]. Their results contrast with the data presented by Zhang et al. (2017) [14].

The negative symptoms themselves are associated with cognitive dysfunction. During our research, we also compared the scores of the PANSS and WCST. Our findings are in accordance with Mellilo et al. (2023), who, in their published systematic review, revealed a significant connection between the severity of negative symptoms and cognitive functioning [63].

Even though for many years, the positive symptoms of schizophrenia were the main aim of the pharmacological industry, as far as the cognitive functions are concerned, many of the studies prove no significant correlation between them [64,65,66,67]. Our results support their conclusions. However, such a premise is not validated by the paper by Ruiz-Castanera et al. (2023), which states that positive symptoms influence cognitive and emotional functions [68].

Correlation between negative symptoms and serum lipid levels was also evident in some previous studies. We found two different significant associations between a PANSS N score and lipid levels. The total cholesterol level was negatively correlated with the severity of negative symptoms at the beginning of the experiment, and a lower HDL level was indicative of more prominent negative symptoms at the 8th week of testing. Kim et al. (2019) found a similar correlation between HDL and PANSS negative subscale results [69]. Our result is partially in alliance with Goldsmith et al. (2021) results, which detected a significant connection between a PANSS negative subscale score and total cholesterol levels as well as LDLs [70].

In our study, we did not reveal any differences in WCST scores concerning antipsychotic treatment, possibly due to a naturalistic approach, treating patients with different antipsychotics, often not in monotherapy, and with adjuvant other pharmacological agents, such as benzodiazepines and antidepressants patient groups. A short (8 weeks) clinical observation could also influence the obtained results. The effect of antipsychotics on cognitive functioning in schizophrenia is not well understood. Antipsychotics may partially improve cognitive functioning, and this improvement may vary depending on the class of the antipsychotic agent, and the specific cognitive domain. There is a large amount of research on the effect of first- and second-generation antipsychotics on cognitive functions in schizophrenia. However, results from the Clinical Antipsychotic Trials of Intervention Effectiveness (CATIE) study indicated that antipsychotics are very similar in their action across chemical classes, and the effect size for improvement in cognition was small, with questionable clinical significance [71].

### Limitations

There are several limitations in the generalizability of our results. Firstly, the size of our sample was relatively small, and the sample itself was homogenous in terms of gender (only women were recruited for this study). Secondly, there was a lack of an age-matched control group. Furthermore, the patients were treated with various antipsychotic agents, which might have influenced the results. In addition, we only used the WCTS as a measurement for cognitive functioning; in the future, the use of standardized batteries of tests (e.g., MATRICS) might enable more accurate cross-study comparisons.

## 5. Conclusions

In summary, our research found a significant correlation between cognitive functioning, lipid serum levels, and biomedical variables in schizophrenic patients. Our results suggest a high HDL level might be considered a protective factor against cognitive impairment. In contrast to other studies, we found no correlation between total cholesterol serum levels and cognitive impairment. The influences of age and illness duration also played a vital role in cognitive performance. While taking into consideration our findings, the severity of negative symptoms seemed to strongly influence the cognitive performance of schizophrenic patients, whereas positive symptoms were not proven to be a significant factor in cognitive test results. We are aware that these results should be interpreted with caution due to the limitations of our study. However, our results seem promising for further studies conducted with larger samples, which should further explore the potential influence of serum cholesterol levels, especially HDL levels, and biomedical factors on the cognitive functioning of patients suffering from schizophrenia.

## Figures and Tables

**Table 1:** Clinical characterization and lipid profile of the study group.

Variable	
Age (mean ± SD)	32.96 (±10.90)
Age at onset (mean ± SD)	24.85 (±6.50)
Illness duration (mean ± SD)	8.12 (±8.69)
Recent onset (≤5 years)/chronic (>5 years) (*n*)	30/22
BMI T0 normal (≥25)/higher (<25) (*n*)	43/9
Smoking yes/no (*n*)	20/32
Normal/high LDL (*n*)	22/30
Normal/low HDL (*n*)	41/11
Normal/high TG (*n*)	41/11
Low-density lipoprotein LDL T8 (mg/dL)	120.5 (±36.19)
High-density lipoprotein HDL T8 (mg/dL)	64 (±14.47)
Triglycerides (TGs) T8 (mg/dL)	119 (±56.44)
WCST trials administered (mean ± SD)	108.10 (±22.15)
WCST total correct (mean ± SD)	65.73 (±15.35)
WCST % errors (mean ± SD)	36.06 (±19.67)
WCST % perseverative responses (mean ± SD)	23.85 (±21.22)
WCST % perseverative errors (mean ± SD)	20.48 (±16.05)
WCST % nonperseverative errors (mean ± SD)	15.54 (±12.92)
WCST % conceptual level responses (mean ± SD)	54.21(±26.19)
WCST categories completed (mean ± SD)	4.23 (±2.22)
WCST trials to complete 1st category (mean ± SD)	29.63 (±38.84)
WCST failure to maintain set (mean ± SD)	0.88 (±1.22)
WCST learning to learn (mean ± SD)	−5.41 (±10)
PANSS positive T0 (mean ± SD)	21.83 (±4.42)
PANSS negative T0 (mean ± SD)	24.48 (±5.03)
PANSS general T0 (mean ± SD)	39.13 (±7.09)
PANSS total T0 (mean ± SD)	85.44 (±13.14)
PANSS positive T8 (mean ± SD)	11.29 (±2.61)
PANSS negative T8 (mean ± SD)	18.77 (±5.07)
PANSS general T8 (mean ± SD)	27.54 (±5.61)
PANSS total T8 (mean ± SD)	40.31 (±7.87)

**Table 2:** Comparisons of Wisconsin Card Sorting Test (WCST) results, Positive and Negative Syndrome Scale (PANSS), and lipid profile with regard to disease onset (recent (≤5 years) vs. chronic (>5 years)).

Variable	U	Z	*p*
WCST trials administered	305.0	−0.49	0.627
WCST total correct	171.5	2.93	0.003
WCST % errors	246.5	−1.54	0.124
WCST % perseverative responses	233.5	−1.78	0.075
WCST % perseverative errors	218.5	−2.06	0.039
WCST % nonperseverative errors	309.0	−0.38	0.704
WCST % conceptual level responses	251.5	1.45	0.148
WCST categories completed	266.5	1.25	0.211
WCST trials to complete 1st category	319.5	−0.19	0.851
WCST failure to maintain set	229.5	2.02	0.044
PANSS positive T0	324.5	−0.00	0.993
PANSS negative T0	281.5	−0.81	0.416
PANSS general T0	272.5	−0.98	0.327
PANSS total T0	277.5	−0.89	0.375
PANSS positive T8	300.5	0.46	0.645
PANSS negative T8	309.0	−0.3	0.765
PANSS general T8	297.0	−0.52	0.600
PANSS total T8	312.0	−0.24	0.808
LDL T0	219.0	−1.43	0.151
HDL T0	279.5	0.20	0.839
TG T0	271.0	−0.65	0.516
LDL T8	219.0	−1.43	0.151
HDL T8	273.5	0.96	0.337
TG T8	282.0	−0.80	0.423

Mann–Whitney U-test.

**Table 3:** Analysis of Wisconsin Card Sorting Test results and PANSS T8 scores with regard to high-density lipoprotein (HDL) levels (normal vs. low).

Variable	U	Z	*p*
WCST trials administered	120.5	−2.504	0.012
WCST total correct	156.5	1.537	0.124
WCST % errors	104.5	−2.702	0.007
WCST % perseverative responses	143.5	−1.829	0.067
WCST % perseverative errors	143.5	−1.831	0.067
WCST % nonperseverative errors	97.5	−2.863	0.004
WCST % conceptual level responses	107.5	2.634	0.008
WCST categories completed	113.5	2.677	0.007
WCST trials to complete 1st category	141.5	−1.896	0.058
WCST failure to maintain set	208.5	−0.403	0.687
WCST learning to learn	93.5	0.766	0.444
PANSS P8 total	155.5	−1.571	0.116
PANSS N8 total	131.0	−2.111	0.035
PANSS G8 total	174.0	−1.147	0.251
PANSS 8 total score	166.0	−1.324	0.185

Mann–Whitney U-test.

## Data Availability

The original contributions presented in the study are included in the article/Supplementary Materials, further inquiries can be directed to the corresponding authors.

## Supplementary Materials

The following supporting information can be downloaded at: https://www.mdpi.com/article/10.3390/brainsci14070699/s1, Table S1: WCST performance with regard to pharmacological treatment.
